# Serum zinc levels and body composition variability as trajectory for hyperlipidemic and dyslipidemic effect among welders exposed to welding fumes and smoking: A biomarker for cardiovascular health

**DOI:** 10.1016/j.toxrep.2024.05.008

**Published:** 2024-05-22

**Authors:** Bartholomew Chukwuebuka Nwogueze, Mary Isioma Ofili, Kenneth Kelechi Anachuna, Alphonsus Okafor Mbah

**Affiliations:** a Physiology Department, Delta State University, Abraka, Delta State, Nigeria; b Nursing Science Department, Delta State University, Abraka, Delta State, Nigeria

**Keywords:** Welder, Total cholesterol, Triglyceride, High-density lipoprotein, Low-density lipoprotein, Body mass index, Cigarette smoke

## Abstract

Welding is a common method for joining metals by heating them to the welding temperature. Exposure to welding fumes has a serious effect on the health of welders. This study examined serum zinc variability and body composition as route for hyperlipidemia and dyslipidemia in welders exposed to welding fumes and smoking, exploring the possibilities for the risk of possible cardiovascular disease. The experimental case control design was adopted in the study. Forty apparently healthy adult males were randomly selected comprising of twenty control group (non-smokers and smokers without welding experience) and twenty experimental group (non-smokers and smokers with welding experience) welders. Data obtained were represented as Mean ± SEM while comparison of means across group was done by one-way ANOVA followed by Tukey's multiple comparison for post hoc test at p-value < 0.05 level of significance using Graph Pad prism version 8. The data obtained showed that the body mass index (BMI) of smokers (non-welders and welders) were slightly reduced while that of non-smoking welders was increased compared to the control. The serum zinc level increased among the smoking welders, while the smoking non-welders and non-smoking welders decreased when compared to the control group (p < 0.05). Exposure to welding fumes has been shown to increase total cholesterol levels compared to the control. Weld fumes significantly (p < 0.05) increased high-density lipoproteins (HDL) levels among smoking non-welders compared to the control group, while, HDL was reduced in non-smoking welders and smoking welders, respectively (p < 0.05). Triglyceride levels significantly (p < 0.05) increased in all experimental groups compared to control levels (p < 0.05). Exposure to welding fumes and smoking caused significant changes in serum zinc, HDL and triglycerides levels with implications for the formation of plaques around the arteries interfering with the effective flow of blood through the vascular system, with implications of hyperlipidemia and dyslipidemia. This study recommends that further studies should be done using biomarkers from urine or toe nails.

## Highlights

• Serum zinc levels and body composition variabilities as trajectory.
• Hyperlipidemic and dyslipidemic effect among welders exposed.
• Exposure to welding fumes and smoking.
• Biomarker for cardiovascular health.
• Welding fumes increased the levels of high density lipoproteins and triglyceride.

## Graphical Abstract

**Figure:**
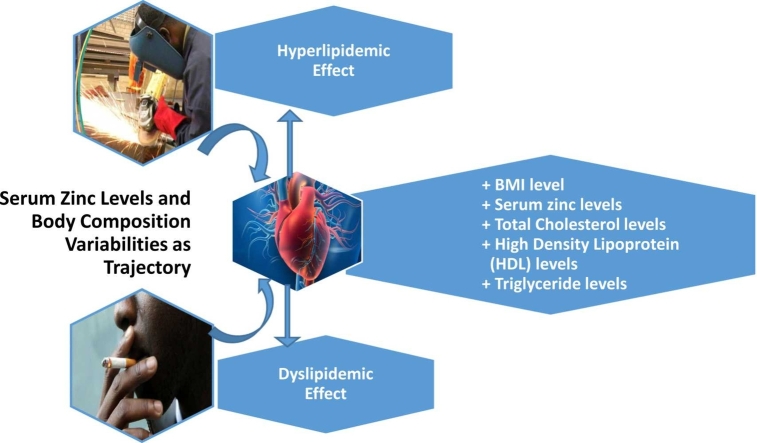

## 1 Introduction

Welding has been a noble occupation from since the earliest times. Welding is a common industrial process, so common that up to 2% of the working population in industrialized countries has engaged in some sort of welding. However, welding is also a hazardous process [1]. Welding is the most important industrial process for joining metals, but at the same time it is a significant source of poison smoke and gas emissions [2]. Fumes emanating from welding processes are airborne metal particles produced under high heat. In other words, welding fumes are a complex mixture of metallic oxides, fluorides, and silicates generated by the evaporation and subsequent condensation of hot metal vapours during welding [3]. Welding fumes consist of metal oxide particles and gases that are formed during welding. The composition and size of the particles depend on the selected welding process as well as on the base and filler materials. The particles are small enough to remain in the atmosphere as airborne particles that are often inhaled [4].

Welding is a common process that is used to join metals by heating them to the welding temperature. Welding processes produce hazardous agents including fumes, gases, vapours, heat, noise, and ultraviolet and infrared radiation [3]. The composition the electrode and the material being welded play a major role in determining the elemental make up of a given welding fume [5]. Welders are exposed to a wide range of metals and nonmetals with varying and sometimes additive value toxic effects. Grishagin et al. [5] state that metallic particles are susceptible to wind-borne impacts and may disperse outside of the work area prior to being absorbed by the welder's body. As such, fumes generated during the welding process are believed to be the most hazardous when compared to other welding by-products. According to Kauppi et al. [6], there is a consistent correlation between the concentration of particles inside the breathing spaces and the results inside the welding face shields.

Welding poses health threats not only to humans but also the environment. This is not entirely from the toxic metals only, but also due to welding fumes containing toxic substances such as carbon monoxide, carbon dioxide, phosgene, asbestos hydrogen fluoride, and nitrogen oxides [7], [8], [9]. Welding fumes have been affirmed by a previous studies to induce adverse health effects, such as neurological, respiratory, venotoxic, gastrotoxic and other health-related problems, such as; various inflammatory processes linked to central nervous system disorders [10], [11], infection of primary organs like; heart, lung, and larynx [12], and urinary tract cancers [13]. Human health risks due to exposure to welding fumes are associated, as a rule, with multiple factors such as metallic content, smoking, etc [4].

Zinc, an essential trace element, is reported to be involved in the synthesis, storage, and secretion of insulin and has been reported to play a vital role in several physiological processes such as the catalytic reaction of enzymes, cell growth, differentiation, and modulation of nucleic acid structure [14]. It is a cofactor for the activity and folding of proteins [10]. Zinc is represented by the symbol “Zn”, with an atomic number and a weight of 30 and 65.38 g, respectively. It was discovered and recognized as a metal for the first time in 1746 by the German chemist Andreas Sigismund Marggraf. Zinc is a significant occupational heavy metal toxin, especially within the metal processing industries. Due to the pleiotropic effect of zinc on all aspect of cell physiology, zinc deficiency or excessive increase in its cellular concentration can and are catastrophically related to important pathophysiology such as diabetes and stroke [15], [16]. Zinc-containing welding fumes appear in large quantities in the production of brass, galvanized metals, and various other alloys. Acute metal fume fever is commonly associated with zinc inhalation by welding, electroplating, oxygen cutting or plating of brass, dyeing, electroplating and galvanizing processes [17]. Zinc has now become a focus of considerable attention in cardiovascular health and cardiovascular disease epidemiology. However, its role has not yet been fully understood. Exposure to these fumes is known to cause metal fume fever and other health associated pathologies like; chills, nausea, throat dryness, cough, fatigue, general weakness, aching of the head and body, vomiting, and dyspnea. Dyslipidemia, which occurs secondary to obesity and is a primary risk factor for coronary and cerebrovascular disease, has also been inversely linked to zinc levels in various cross-sectional and clinical studies. Hence, workers need adequate protection from vapors produced during metal work [18].

Body mass index (BMI) is a value derived from a person’s mass (weight) and height. BMI is defined as body mass divided by the square of height and is expressed in the unit kg/m^2^, which results from the mass in kilograms and the height in meters [19]. BMI is a handy rule of thumb used to roughly categorize a person as underweight, normal weight, overweight or obese based on tissue mass (muscle, fat, and bone) and height [20]. The main BMI classifications for adults are underweight (below 18.5 kg/m^2^), normal weight (18.5–24.9 kg/m^2^), overweight (25–29.9 kg/m^2^) and obese (30 or more kg/m^2^) [21]. When used to predict an individual’s health rather than as a group statistical measure, BMI has limitations that may make it less useful than some of the alternatives, particularly when applied to individuals with abdominal obesity, short stature, or abnormally high muscle mass. BMIs below 20 and above 25 kg/m^2^ were associated with higher all-cause mortality, with the risk increasing with distance from 20 to 25 kg/m^2^ [21], [22].

The lipid profile, also referred to as lipid panel, is the collective term given to the estimation of typically, total cholesterol (TC), high-density lipoprotein (HDL), low-density lipoprotein (LDL), and triglycerides; they are considered essential to the human, both because they make up the fundamental structure of the cell membrane and because they act as a precursor to steroid hormones, bile acids, and vitamin D [23]. Welders exposed to fumes should be subjected to lipid panel, which is a blood test that serves as an initial broad medical screening tool for abnormalities, mostly seen in obesity [24]. Lipid profile biomarkers include; triglycerides and total, high-density lipoproteins, low-density lipoproteins and cholesterol are modifiable factors sensitive to obesity [25]. Lipid profiles are measures of global risk assessment, and the frequency of check-ups is determined by age, sex, and risk factors for cardiovascular diseases. It is vital to estimate the probability of particulate matter being constituents of welding fumes and its related health imparting on the function of the lipid profile. Such variability entails that fasting lipid profile is the most precise lipid assessment, specifically triglycerides, while non-fasting lipid profile provides accurate measurement of other lipids including total cholesterol, and HDL cholesterol, respectively. It is critical to better understand the possible adverse health effects of exposure to welding fumes, especially as it concerns hyperlipidemia and dyslipidemia and the possible underlying mechanisms involved in assessing risk and creating preventive measures that will benefit a significant workforce [26].

Smoking is one of the environmental factors that can alter the normal lipid profile. It is one of the risk factors causing coronary atherosclerosis, inflammation, alters immune function, and the development of coronary artery diseases [27]. Cigarette smoking has another negative impact on serum lipid profile levels. The lipid profile plays a vital role in human health. Some of the roles include serving as hormone or hormone precursors, storage function, helping in digestion, providing energy and metabolic fuels; acting as structural and functional compounds in bio membranes in forming insulation to allow nerve conduction or prevent heat loss [27]. Cigarette smoking is a complex blend of more than 4,800 components identified that contain high levels of free radicals, reactive oxygen and nitrogen species, reactive aldehyde, and diverse metals [28]. Recent studies support the hypothesis that exposure to cigarette smoke increases oxidative stress as a potential mechanism for initiating cardiovascular dysfunction. Smoke quickly damages blood vessels throughout the body and makes blood more likely to clot. Chemicals in tobacco smoke damage the delicate lining of the lungs and can cause permanent damage that reduces the ability of the lungs ability to exchange air efficiently. This can ultimately lead to chronic obstructive pulmonary diseases, including emphysema. Meanwhile, nicotine and other toxic components of tobacco cigarette smoke are absorbed through the lungs into the bloodstream and circulate throughout the body [28].

Welders are risk groups for cardiovascular disease because inhalation toxicants can also affect the cardiovascular system. Given the established detrimental impacts caused by welding fume exposure on cardiovascular health and lipid profiles, it is critical to find potential biomarkers or effect predictors. A marker like this is serum zinc. Due to its function in antioxidant enzyme systems, low serum zinc levels are recognized as an indicator of cardiovascular disease and death. Elevated zinc levels have been shown to increase the risk of cardiovascular disease [28]. Hence, it becomes imperative to understudy the effect of welding fume exposure and attitude to smoking on the variability of serum zinc and lipid profile (cholesterol, HDL, and triglycerides) and body mass index of welders with possible risk of cardiovascular diseases.

## 2 Methods

### 2.1 Materials used

The following materials and instruments will be used in the study: stadiometer, weighing scale, methylated Spirit, cotton wool, 5 ml syringe and needle, blood test tubes (plain), hand gloves, electronic weighing balance, bucket hematocrit centrifuge, sterile bottles and containers.

### 2.2 Research participants

Forty (40) apparently healthy male adults comprised of welders and non-welders that were within the ages of 18–65 years. The welders and non-welders residents of Abraka in the Ethiope East Local Government Area and Obiaruku in the Ukwuani Local Government Area, both of Delta State, Nigeria respectively, were carefully selected and recruited as the target participants for the study.

### 2.3 Sample and sampling technique

All the total 40 participants comprising 20 participants from Abraka and 20 participants from Obiaruku respectively were carefully selected as the sample for our study. Convenient nonprobability sampling technique was employed to locate the welders, but simple random sampling technique. Balloting without replacement was utilized to select study participants. 40 ballots each of ‘Yes’ and ‘No’ were presented to those who consented to be qualified to be recruited for the study; however, only subjects that picked ‘Yes’ were considered study participants.

### 2.4 Research design

This study used a case-control experimental research design to examine variability in serum zinc levels, lipid profile, and body mass index among welders exposed to welding fumes and smoking. It consisted of the control and experimental groups as described below;

1. **Control group** (n = 20): Comprised of non-smoking and smoking individuals that are not welders. The control group has the following subgroups:

• **Negative control group I** (n = 10): individuals whose occupational description is not related to welding, or exposed to welding fumes and do not reside close to any welding shop(s) and do not smoke.
• **Positive control group II** (n = 10): individuals whose occupation is not welding, but apparently smoke.

2. **Experimental group** (n = 20): Comprised of welders who are non-smokers and smokers respectively. The experimental group will have the following sub-groups:

• **Experimental group I** (n = 10): Smoking welders who are exposed to welding fumes
• **Experimental group II** (n = 10): Non-smoking welders who are exposed to welding fumes

### 2.5 Inclusion and exclusion criteria

The study recruited only welders were in the age range of 18–65 years of age who resided in Obiaruku and Abraka. Only those who have worked at least one year as welders. Apparently healthy adults who did work in industries dealing with aluminum or metal fabrications were selected. The category of subjects not included in the study was those who are paralyzed or have a history of trauma, fracture, arthritis, neurological conditions, hypertension, respiratory and cardiovascular related problems.

### 2.6 Procedure for data collection

In the study process, it was observed that the welders resident in Obiaruku have a union that guides their operations in the region, but the researchers were not notified earlier about the actual location and time of convergence for their monthly meetings, so the researchers had to tour their individual workshops and locations. In the Abraka region, there was no unified body that guided the welders, so the researcher had to meet them at their various locations with the help of a resident welder who identified himself with them. The aim and the procedures for the study was explained to the participants and signed informed consent was sought and obtained. The researcher collected blood samples (5 ml) from each participant. To estimate the BMI, the heights of the participants were obtained using a centimeter calibrated stadiometer. The stadiometer was placed on a flat surface and participants were asked to remove their shoes and stand on the stadiometer in an upright position with their heel in contact with the vertical bar of the stadiometer and the readings were recorded. For the body weight, the participants were instructed to step on a weighing scale with bare foot, standing erect , and looking straight at an eye level; while the reading was taken immediately.

### 2.7 Biochemical examination

#### 2.7.1 Serum zinc analysis

Serum zinc levels was measured using the techniques of Xinhua et al. [29]. The samples were read on Buck Scientific atomic absorption spectrophotometer (AAS) model 210/211 VGP. The metal was read at a desired wavelength and the metallic concentration in each sample was displayed on the digital read out. Considering zinc wavelength and lamp, the samples were read using an acetylene gas to ignite the machine.

#### 2.7.2 Estimation of serum total cholesterol

Cholesterol is a form of fat (lipid) that is found in the body that can be detected in both serum and blood plasma. It can exist in both free and esterified forms. Cholesterol esterase is capable of hydrolyzing cholesterol ester to produce free fatty acids and cholesterol. Cholesterol oxidase breaks down cholesterol to create hydrogen peroxide and 4-cholestenone. Red quinone compounds of benzoquinone imine phenizon are formed when hydrogen peroxide catalyzes peroxidase in the presence of 4-aminoamylpyridine and phenol. The amount of cholesterol is directly correlated with the depth of color of the quinone produced. The relationship between the color intensity and serum concentration is linear. It is possible to compute the cholesterol content of the sample using the numbers obtained from the absorbance standard tube and sample tube measurements [30].

#### 2.7.3 Estimation of serum high-density lipoproteins (HDL)

High-density lipoproteins values are also used in the calculation of low-density lipoproteins (LDL) using the direct HDL method, although, HDL is measured directly in serum using the method adopted by Naiho *et al.,* [31]. The basic principle of the method is as follows. The basic principle of the procedure is as follows. The apoB-containing lipoproteins in the samples are reacted with a blocking reagent that makes them unresponsive to the enzymatic cholesterol reagent under the conditions of the assay. The process uses sulfated alpha-cyclodextrin in the presence of Mg^2+^ that forms complexes with apoB lipoproteins, and polyethylene glycol-coupled cholesteryl esterase and cholesterol oxidase for the measurement of HDL cholesterol.

#### 2.7.4 Estimation of serum triglycerides

Triglycerides are measured enzymatically in serum using a series of coupled reactions in which triglycerides are hydrolyzed to produce glycerol using the method adopted by Naiho et al., [31]. Glycerol is then oxidized with the use of glycerol oxidase, and H_2_O_2_, one of the reaction products, is measured as illustrated above for cholesterol. The absorbance is measured at 500 nm.

#### 2.7.5 Data analysis

The data were subjected to descriptive statistics and analyzed using paired data analyzed by one-way analysis of variance (ANOVA). Tukey’s multiple comparison tests was used to test for Post hoc association between variables. Graph pad prism version 8 was used to compute the data. Significance is measured at P < 0.05. Figure legend that contain * implies significantly different from the control group; ‘a’ implies significantly different from the smoking non-welders group; ‘b’ implies significantly different from non-smoking welders group; and ‘c’ implies significantly different from smoking welders group.

## 3 Results

**Fig. 1:**
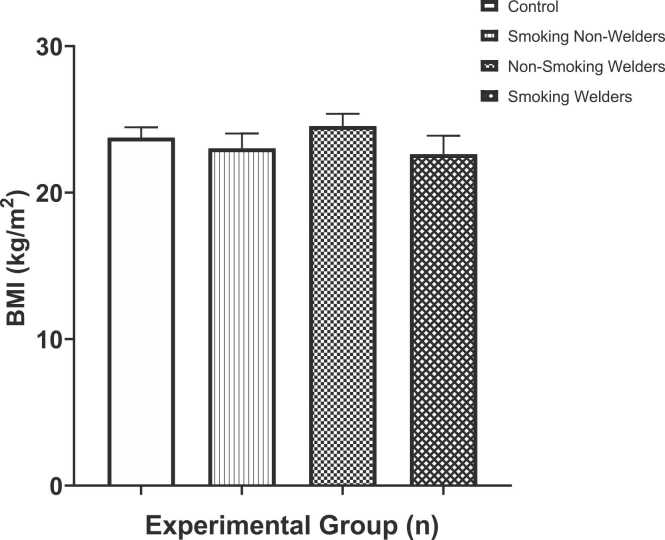
Represents the body mass index (BMI) of workers exposed to welding fumes and smoking.

The body mass index participants who are exposed to smoking and welding fumes is shown in Fig. 1. The mean BMIs of the experimental groups; smoking non-welder (23.0 ± 1.01 kg/m^2^), non-smoking welders (24.5 ± 0.84 kg/m^2^) and smoking welders (22.6 ± 1.26 kg/m^2^) did not differ significantly the control group (23.8 ± 0.71 kg/m^2^) according to the results. In our study, smoking non-welders and smoking welders respectively had a low BMI level when compared to the control group. However, the BMI level was increased in non-smoking welders when compared to the control group and when compared between the experimental groups. The one-way ANOVA test for multiple comparison among means revealed that there was no significant difference in the values of BMI (F [3,36] = 0.768, p < 0.52).

**Fig. 2:**
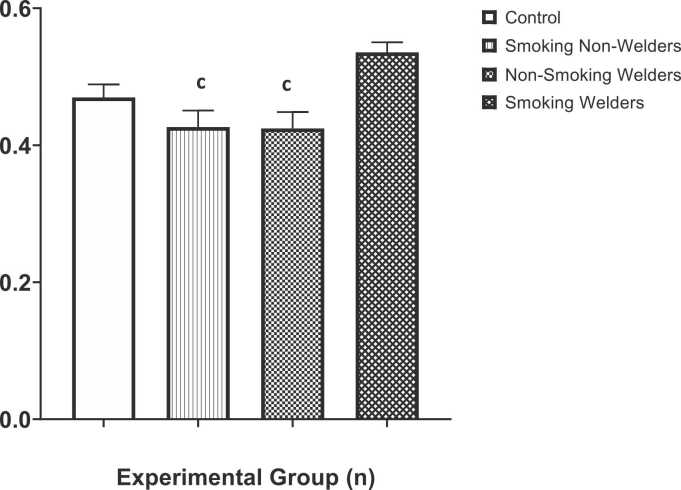
Evaluation of serum zinc levels in workers exposed to welding fumes and smoking. c: Significantly different from smoking welders group.

The results of the serum zinc levels of participants who were exposed to smoking and welding fumes are shown in Fig. 2. Compared to the mean serum of the control group (0.47 ± 0.02 μg/dL), participants who are smoking non-welders (0.43 ± 0.02 μg/dL) and non-smoking welders (0.43 ± 0.02 μg/dL) had a very low mean serum zinc levels; however, the observed decrease was not statistically significant from the control level, but was statistically significantly (p < 0.05) different compared to the mean serum zinc levels of the smoker welders (0.54 ± 0.01 μg/dL) that was increased compared to control, compared to experimental groups (smoking non-welders and non-smoking welders), respectively. The one-way ANOVA test for multiple comparison among means revealed that there was a significant difference in the serum zinc levels (F [3,36] = 6.29, p < 0.0015).

**Fig. 3:**
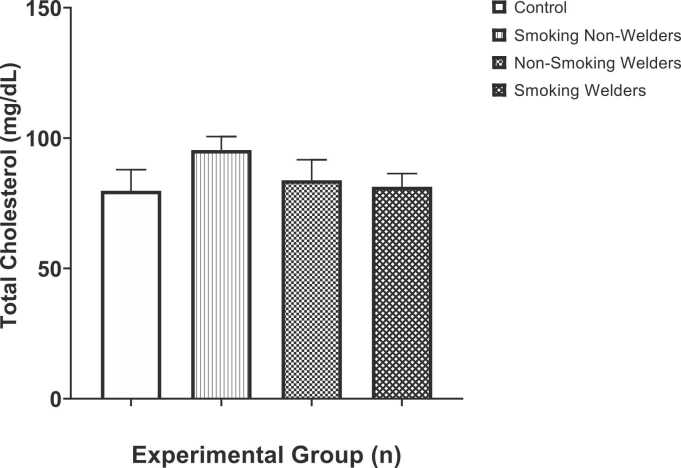
Evaluation of total cholesterol levels in workers exposed to welding fumes and smoking.

Total cholesterol levels in participants exposed to smoking and welding fumes are shown in Fig. 3. Compared to the mean total cholesterol levels of the control groups (79.8 ± 8.11 mg/dL), our study found that the mean total cholesterol levels of smoking welders (95.4 ± 5.21 mg/dL), non-smoking welders (83.8 ± 7.85 mg/dL), and non-smoking non-welders (81.3 ± 5.07 mg/dL) increased (but not significantly). One-way ANOVA test for multiple comparison among means revealed that there was no significant difference in total cholesterol levels (F [3,36] = 1.11, p < 0.3578).

**Fig. 4:**
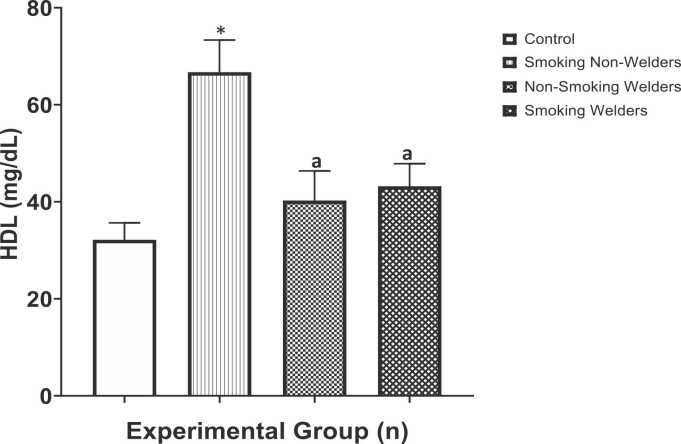
Evaluation of high-density lipoprotein (HDL) levels in workers exposed to welding fumes and smoking. *: Significantly different from the control group. a: Significantly different from the smoking non-welders group.

Fig. 4 presents the results of high-density lipoprotein (HDL) levels in participants exposed to welding fumes and smoking. This study recorded a significant increase (p < 0.05) in the mean HDL levels among smoking non-welders (66.7 ± 6.63 mg/dL) compared to the mean control levels (32.2 ± 3.50 mg/dL) and compared to the experimental groups (non-smoking welders and smoking welders) respectively. Furthermore, mean HDL levels were increased among non-smoking welders (40.3 ± 6.11 mg/dL) and smoking welders (43.2 ± 4.65 mg/dL) compared to the control levels, however, the increase was not up to the levels of smoker non-welders. One-way ANOVA test for multiple comparison among means revealed that there was significant difference in the high-density lipoprotein levels (F [3,36] = 7.65, p < 0.0004).

**Fig. 5:**
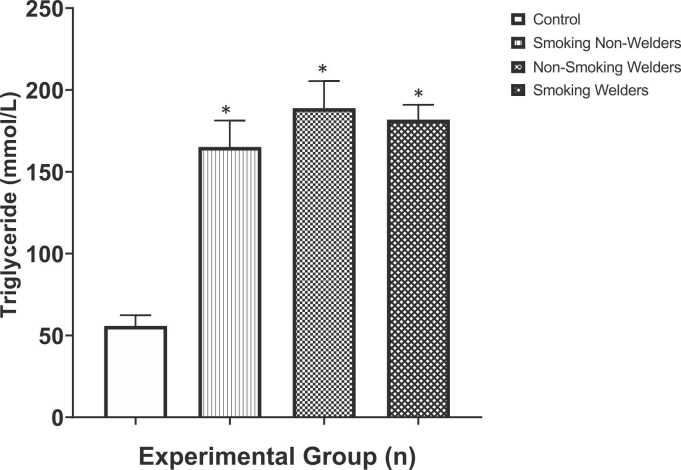
Evaluation of triglyceride levels in workers exposed to welding fumes and smoking. *: Significantly different from the control group.

The results of triglyceride levels in participants exposed to smoking and welding fumes are shown in Fig. 5. Compared of the mean triglyceride levels of the control groups (55.79 ± 6.55 mmol/L), this study found that mean triglyceride levels of smoking non-welders (165.1 ± 16.16 mmol/L), non-smoking welders (188.9 ± 16.57 mmol/L) and smoking welders (181.9 ± 9.05 mmol/L), respectively were statistically significantly (p < 0.05) increased. However, comparison between experimental groups did not show any appreciable variations. One-way ANOVA test for multiple comparison among means revealed that there was significant difference in the triglyceride levels (F [3,36] = 23.43, p < 0.0001).

## 4 Discussion

During the welding process, metal vapours gradually form at extreme temperatures (> 4000 °C) and subsequently cool and condense into fumes [32]. Fumes originating from welding contain hazardous agents which include heat, toxic substances, particulate matter (PM), ultraviolet & infrared radiation, and volatile organic compounds (VOC's); which in turn causes emission of gases [2], [3], [10]. The body mass index (BMI) findings as shown in Fig. 1 revealed that the BMI was reduced in non-welders and welders who smoke compared to the control group. However, the BMI of the non-smoking welders was slightly increased compared to the control. This finding is consistent with the research work done by Perdana *et al.*, [33] on welding fumes exposure, body mass index and duration of smoking decrease physical fitness among welders. Their result showed that the overall increase in exposure to welding fumes, the body mass index, and the duration of smoking behavior will decrease the physical fitness of welders. On the contrary, Rizvi and Nagra, [34] argued that smoking results in increased BMI and lipid profile are strongly associated with cardiovascular heart disease.

Zinc (Zn) is one of the most important metals used in the production of brass, galvanized metals, and other alloys [35]. Fig. 2 shows that there was an increase in serum zinc levels among smoking welders when compared to the control, and compared with serum zinc levels of smoking non-welders, and non-smoking welders respectively. This is similar with the reports of a previous study that investigated the relationship between welding fume exposure, smoking and pulmonary function in welders and found that smoking has a synergistic effect in combination with exposure to welding fumes in the event of pulmonary declination [36]. As such, another study asserts that exposure to zinc-containing welding fumes can induce adverse health effects such as neurological disorders, respiratory and cardiovascular problems [37]. Inhaling zinc oxide fumes causes inflammatory changes in the lungs of man. There is an increased chance of pneumococcal infection and immune suppression in welders [38], and the risk is greater with great influence on welders who smoke, exacerbating higher chances of pneumococcal invasion [39].

Smoking is one of the environmental factors that alter the normal lipid profile. Recent studies support the hypothesis that exposure to cigarette smokes increases oxidative stress as a potential mechanism for initiating organ system alterations like; reproductive, renal, cardiovascular dysfunction [40], [41], [42], [43]. For example, smoke quickly damages blood vessels throughout the body and increases the likelihood of blood clotting. The chemicals within tobacco smoke damage the sensitive lining of the lungs, reducing the ability of the lungs to exchange air efficiently and can cause permanent damage to the walls of blood vessels, which cause plaques [27], [44]. This can ultimately lead to chronic obstructive lung disease, including emphysema [44]. Considering that lipid profile plays vital role in human’s health, some of the roles of which include serving as hormone or hormone precursors, storage function, helping in digestion, providing energy and metabolic fuels; acting as structural and functional compounds in bio membranes in forming insulation to allow nerve conduction or prevent heat loss [27]. As such a previous study reported a relationship between smoking and an increase chances of lipid parameters [44].

Our result as contained in Fig. 3 revealed a non-significant increase in the total cholesterol level of smoking non-welder, non-smoking welders and smoking welders respectively when compared with the control group. This finding agrees with that of Rashan et al., [45] that the mean value of total cholesterol in the smoking group was higher than in the non-smoker group. The higher the blood cholesterol level, the greater the risk of developing plaques on the walls of the arteries, including the arteries that supply blood to the heart, called coronary arteries. As such, a total cholesterol concentrations below 200 mg/dL have been considered desirable, while, concentrations greater than 240 mg/dL are referred to as hyperlipidemic [46]. However, epidemiological evidence suggests that the risk of cardiac events decreases when TC levels drop to approximately 150 mg/dL. Also, TC should be less than 180 mg/dL in children. An elevated level of HDL-cholesterol concentrations is protective against coronary heart disease, while reduced HDL-cholesterol concentrations, particularly in conjunction with elevated triglycerides, increase the cardiovascular risk [47].

Variations in serum HDL cholesterol concentrations are associated with an increased risk of coronary heart disease (CHD). Coronary risk increases markedly as HDL concentration decreases from 40 to 30 mg/dL. A value below 35 mg/dL is considered to be low HDL-cholesterol concentration and values above 60 mg/dL is high HDL. Data from our study as shown in Fig. 4 revealed that there was a significant increase in HDL level among smoking non-welder compared to the control group. Similarly, HDL levels were not significantly elevated in welders (for both smokers and non-smokers) compared to the control levels. This agrees with the reports of He *et al*., [48] who reported that a negative impact of cigarette smoking on the risk of cardiovascular disease seems to be mediated through its effects on lipid and lipoprotein metabolism; thus, cigarette smoking in particular affects HDL levels. This is equally consistent with a previous investigation conducted on acute decreases in HDL cholesterol associated with exposure to welding fumes and reported that welding exposure was associated with a decrease in circulating HDL, which may be related to inflammation and pro-atherosclerotic effects of fine dust pollution [49].

The results of our study revealed that the triglyceride levels were significantly increased in smoking non-welders, non-smoking welders and smoking welders, respectively compared to control levels (see Fig. 5). Triglycerides are not a type of cholesterol, but they are part of a lipoprotein panel (the test that measures cholesterol levels). The triglyceride concentration less than 150 mg/dL is considered as normal, whereas, concentrations of 200–499 mg/dL are considered high which can increase the risk of heart disease [50]. High serum triglyceride levels help indicate conditions associated with an increased risk of coronary heart disease (CHD) and peripheral disease atherosclerosis. High triglycerides are associated with an increased risk of CHD patients with other risk factors, such as low HDL cholesterol, some group of patients with elevated concentrations of apolipoprotein B, and patients with forms of LDL that may be particularly atherogenic. A very high level of triglycerides cause in pancreatitis and should be promptly examined and treated [31]. Perhaps, another study maintained that smoking increases the quantity of bad fats such as; total cholesterol, triglycerides and low-density lipoproteins circulating in the blood vessels and decreases the amount of good fat such as high-density lipoproteins [51].

## 5 Conclusion

The research findings show that, compared to the control groups, the experimental groups of welders exposed to welding fumes did not differ significantly in their body mass indices. Serum zinc level was significantly increased among smoking welders when compared to control levels, whereas, it was decreased in smoking non-welders and non-smoking welders respectively. Exposure to welding fumes has been shown to increase the levels of total cholesterol levels, high-density lipoproteins levels non-significantly and triglycerides levels significantly among smoking non-welders, non-smoking welders and smoking welders respectively, with implications for formation of plaques around arteries interfering with effective flow of blood with possible implications for hyperlipidemia and dyslipidemia as indicator of cardiovascular diseases. Hence, the study recommends that personal protective equipment (PPE) should be given to welders (smokers/non-smokers). Then comparison can be done with respect to zinc serum level or the total lipids to ascertain the level to which the PPE protect people from the welding effects.

## Funding

None.

## CRediT authorship contribution statement

**Bartholomew Chukwuebuka Nwogueze:** Writing – review & editing, Writing – original draft, Supervision, Project administration, Conceptualization. **Mary Isioma Ofili:** Methodology, Investigation, Data curation. **Kenneth kelechi Anachuna:** Visualization, Investigation, Data curation. **Alphonsus Okafor Mbah:** Conceptualization.

## Declaration of Competing Interest

The authors declare that they have no known competing financial interests or personal relationships that could have appeared to influence the work reported in this paper.

## Data Availability

Data will be made available on request.
